# Oxygen Concentration Effect on Conductive Bridge Random Access Memory of InWZnO Thin Film

**DOI:** 10.3390/nano11092204

**Published:** 2021-08-27

**Authors:** Chih-Chieh Hsu, Po-Tsun Liu, Kai-Jhih Gan, Dun-Bao Ruan, Simon M. Sze

**Affiliations:** 1 Department of Electronics Engineering, National Yang Ming Chiao Tung University, Hsinchu 30010, Taiwan; cchsu.06g@g2.nctu.edu.tw (C.-C.H.); jameswsalebron@gmail.com (K.-J.G.); rsduty@gmail.com (D.-B.R.); simonsze@faculty.nctu.edu.tw (S.M.S.); 2 Department of Photonics and Institute of Electro-Optical Engineering, National Yang Ming Chiao Tung University, Hsinchu 30010, Taiwan

**Keywords:** conductive-bridge RAM (CBRAM), InWZnO, bilayer switching layer, endurance cycle, defect distribution

## Abstract

In this study, the influence of oxygen concentration in InWZnO (IWZO), which was used as the switching layer of conductive bridge random access memory, (CBRAM) is investigated. With different oxygen flow during the sputtering process, the IWZO film can be fabricated with different oxygen concentrations and different oxygen vacancy distribution. In addition, the electrical characteristics of CBRAM device with different oxygen concentration are compared and further analyzed with an atomic force microscope and X-ray photoelectron spectrum. Furthermore, a stacking structure with different bilayer switching is also systematically discussed. Compared with an interchange stacking layer and other single layer memory, the CBRAM with specific stacking sequence of bilayer oxygen-poor/-rich IWZO (IWZOx/IWZOy, x < y) exhibits more stable distribution of a resistance state and also better endurance (more than 3 × 10^4^ cycles). Meanwhile, the memory window of IWZOx/IWZOy can even be maintained over 10^4^ s at 85 °C. Those improvements can be attributed to the oxygen vacancy distribution in switching layers, which may create a suitable environment for the conductive filament formation or rupture. Therefore, it is believed that the specific stacking bilayer IWZO CBRAM might further pave the way for emerging memory applications.

## 1. Introduction

In recent years, transparent amorphous oxide semiconductors (TAOSs) have attracted much attention in the novel application of electronic devices [1,2] such as thin film transistors [3,4], sensors [5], and memory [6,7,8]. From many options of TAOSs materials, InGaZnO is the most widely studied because of high mobility, excellent reliability and good uniformity. Briefly, in element can provide high carrier concentration and high mobility, while Ga element will inhibit excessive carrier generation and enhance reliability. Besides, Zn element may improve uniformity and amorphous stability. However, Ga is relatively expensive and is a rare element on the earth. It is urgent to find an effective carrier suppressor to replace the Ga element. From many previous studies, tungsten (W) element doped IZO (InWZnO; IWZO) might be a novel candidate to lower manufacturing costs and even exhibit higher bond dissociation energy [9,10,11]. Furthermore, the distribution of oxygen vacancy in thin film affects not only the thin film transistor device, but also the oxide-based memory [12,13,14].

With simple structure, low power consumption, fast switching speed and high density integration, resistive random access memory (RRAM) has gradually become one of the most popular non-volatile memories in the research of memristor [15]. However, the operating mechanism of RRAM has not yet been clarified. In fact, the conductive filament model is the most acceptable theory model, in which the resistance state can be distinguished by the filament’s formation and rupture [16,17,18,19,20,21,22]. On the other hand, the filament model can be further divided into two kinds of operation mode, which are the oxide resistive random access memory (OxRRAM) and the conductive-bridging random access memory (CBRAM). OxRRAM is based on the oxygen vacancy or ion migration, and CBRAM is based on the drift of metal ions within the switching layer by electrochemical reaction. Notably, compared with the OxRRAM, CBRAM with active metal electrodes (like Cu) behaves more advantageously for integrated circuits, such as reducing electron migration and RC delay [23].

In this study, the influence of oxygen concentration in IWZO which was used as the switching layer of CBRAM is investigated. With different oxygen flow during the sputtering process, the IWZO film can be fabricated with different oxygen concentration and different oxygen vacancy distribution [24]. In addition, a stacking structure with different bilayer switching is also systematically discussed. Compared with the interchange stacking layer and other single layer memory, the CBRAM with a specific stacking sequence of bilayer oxygen-poor/-rich IWZO (IWZOx/IWZOy, x < y) exhibits more stable distribution of the resistance state and also better endurance (more than 3 × 10^4^ cycles). Meanwhile, the memory window of IWZOx/IWZOy can even be maintained over 10^4^ s at 85 °C. As a result, it is believed that the specific stacking bilayer IWZO CBRAM might further pave the way for emerging memory applications.

## 2. Materials and Methods

**Figure 1:**
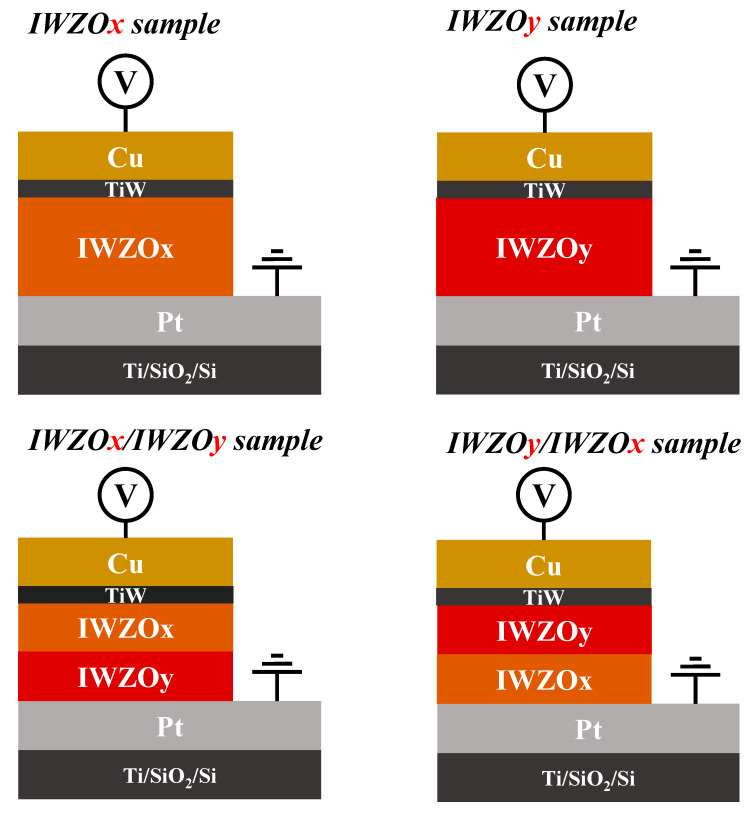
Schematic cross-section diagram of all CBRAM devices with different switching layers.

First, a 100 nm-thick thermal SiO_2_ was grown on the 550 μm-thick p-type Si substrate by furnace. Then, a 5 nm-thick Ti was used as an adhesion layer, and a 100 nm-thick Pt was used as a bottom electrode. Two layers were deposited by the DC sputtering method. After the bottom electrode formation, the switching layer IWZO was deposited on the bottom electrode by the RF sputtering under a based pressure of 4 × 10^−4^ Pa and a working pressure of 0.4 Pa. Afterward, a 2 nm-thick barrier layer TiW and 100 nm-thick Cu top electrode were patterned by circular mask (KEY STAR ELECTRON CO., LTD. Taiwan) with a diameter of 100 μm. For CBRAM devices with a single IWZO layer, the gas flow rate ratio of O_2_/Ar was set as 2/20 and 7/20 during the sputtering process, respectively. On the other hand, the CBRAM devices with bilayer IWZO were deposited with the interchange of different O_2_/Ar gas flow rate ratios of 2/20 and 7/20, respectively. With four Cu/TiW/IWZO/Pt single or stacking structures, the IWZO CBRAM devices were fabricated at room temperature and named IWZOx, IWZOy, IWZOx/IWZOy and IWZOy/IWZOx, respectively, as shown in Figure 1. Furthermore, the thickness of switching layers in four different devices is fixed at 10 nm for comparison.

In terms of material analysis, the cross-sectional image of the device was observed by transmission electron microscopy (TEM), and the surface roughness of thin film was obtained from an atomic force microscope (AFM). In order to further confirm the composition, X-ray photoelectron spectroscopy (XPS) was used for analyzing the detail of the chemical composition. As for the electrical properties, all CBRAM devices were measured by the Keithley 4200 semiconductor analyzer.

## 3. Results

**Figure 2:**
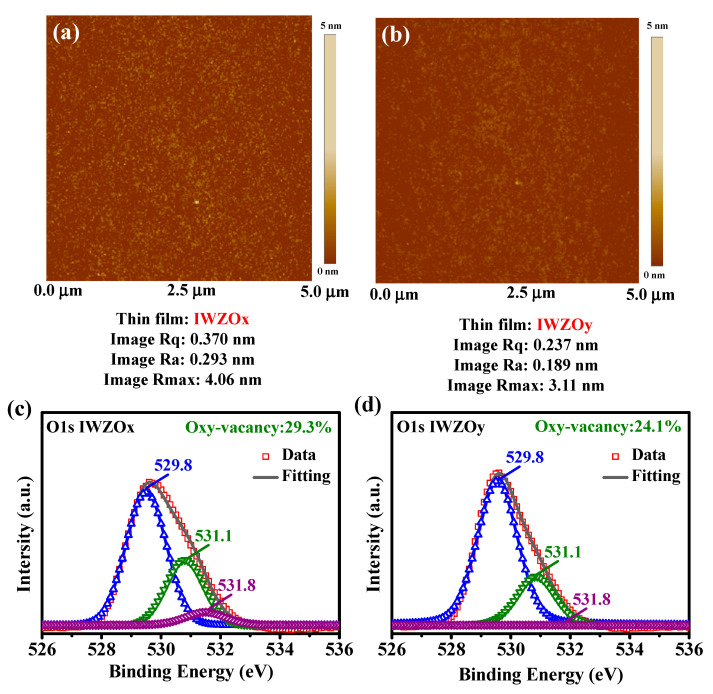
The surface morphologies and Rq of (**a**) IWZOx and (**b**) IWZOy by AFM; Analysis of O1s spectrum for IWZO with different oxygen ratio in process: (**c**) IWZOx and (**d**) IWZOy by XPS.

Generally, the root-mean-square roughness (Rq) may represent the fluctuation of morphologies from reference height and can be extracted by AFM measurement. Figure 2a,b shows the Rq of the IWZO film with different oxygen gas flow ratios in the sputtering process; these are 0.37 nm and 0.24 nm of IWZOx and IWZOy, respectively. Based on the aforementioned results, it can be speculated that lower oxygen gas flow during the sputtering process may induce a higher gas flow ratio of argon, which means that more ion bombardment hit the surface of the IWZO film.

To further investigate the influence of oxygen concentration during IWZO film deposition, O1s peaks obtained by XPS have been deconvoluted into oxygen lattice, oxygen vacancy and oxy-hydroxide, which is corresponded to 529.8 eV, 531.1 eV and 531.8 eV, respectively [10,25]. Figure 2 shows the XPS spectra of (c) IWZOx film and (d) IWZOx film. It is clear that the IWZOx film with a relatively high argon ratio and low oxygen ratio in the sputtering process exhibits more oxygen vacancy than the IWZOy film. This might be because there is more ion bombardment hitting the surface of the IWZO film. Therefore, more defects or oxygen vacancies are generated inside the film, which is consistent with the AFM results shown in Figure 2a. On the other hand, with higher oxygen radical concentration generated from plasma, more active oxygen can be bonded with In atom and form a metal-oxide bond, as obtained from the XPS result of IWZOy film with fewer oxygen vacancies. Besides, lower oxy-hydroxide concentration can also be observed in the IWZOy film, which can be attributed to the surface clean effect during plasma immersion.

**Figure 3:**
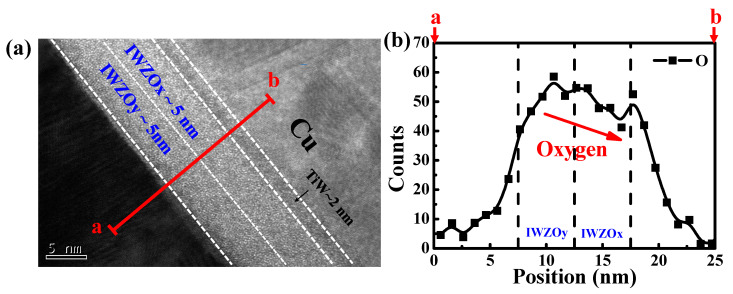
(**a**)The cross-sectional TEM image for a CBRAM device with a stacking IWZOx/IWZOy switching layer. (**b**) Line profile of the O element content along the red line in (**a**) for an IWZOx/IWZOy device.

Figure 3a shows the stacking structure of a CBRAM device with an IWZOx/IWZOy switching layer with a high resolution TEM image. The device structures including Pt bottom-electrode, 5 nm-thick IWZOy film, 5 nm-thick IWZOx film, 2 nm-thick barrier layer TiW and Cu top-electrode are all clearly observed. Furthermore, without obtaining any diffraction, the IWZO switching layer might be kept in an amorphous phase after fabrication. Figure 3b exhibits the EDS line scanning analysis of the O element performed along the red line as labeled in Figure 3a. The oxygen concentration in IWZO film is gradually decreased from Pt to Cu, consistent with the different oxygen content on the bilayer oxygen-poor/-rich IWZO (IWZOx/IWZOy, x < y). 

**Figure 4:**
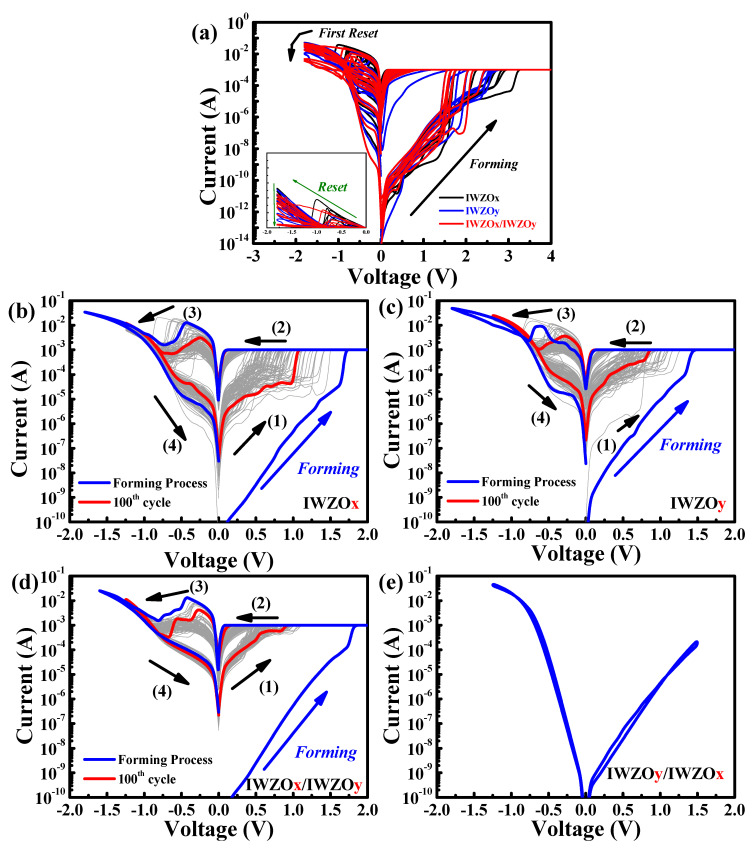
(**a**) Forming and first reset (magnified in inset) process for IWZOx, IWZOy, and IWZOx/IWZOy are obtained from multiple CBRAM devices. Typical bipolar I–V curves for 100 cycles after the initial forming process were measured from the CBRAM devices with the (**b**) IWZOx, (**c**) IWZOy, (**d**) IWZOx/IWZOy, (**e**) IWZOy/IWZOx switching layer.

**Figure 5:**
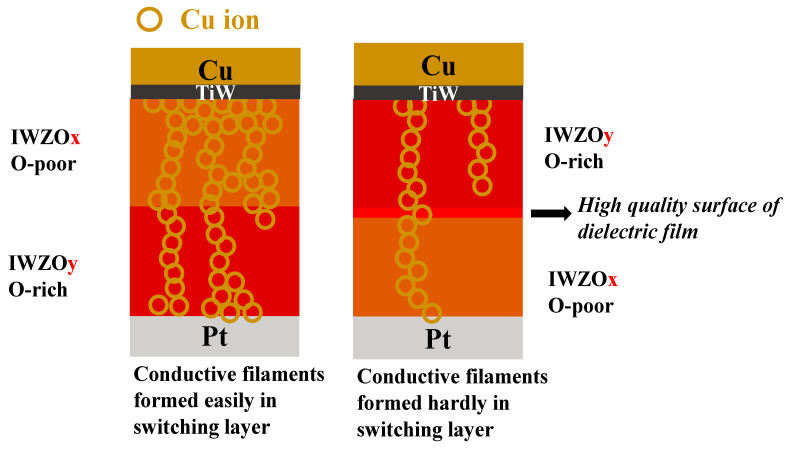
Schematic diagram of the behavior of conductive filaments in the bilayer IWZO CBRAM during device operation.

Figure 4 shows the I-V curve of all the CBRAM devices by voltage sweep mode. In order to initiate the device, a larger voltage bias needs to be applied on the new device. This is referred to as the forming process. At the same time, an appropriate compliance current limit (10^−3^ A) is applied to prevent the hard breakdown of the memory device. After the forming process, a positive voltage is applied on the Cu electrode to switch the resistance state to low (LRS), which is defined as process (1) as shown all Figure 4. Figure 4a shows the forming and first reset process of the CBRAM devices with IWZOx, IWZOy, IWZOx/IWZOy (x < y) switching layers. The forming voltages of the IWZOx/IWZOy device range from 1.2 V to 2.1 V, which is more concentrated than IWZOx and IWZOy devices. In addition, when the device executed the first reset process, it was observed that the current of IWZOx/IWZOy device is smaller and more uniform than other devices [26,27]. During the process (1), a conductive filament is formed with the dissociated Cu ion between the Cu and Pt electrode. On the other hand, this conductive filament will be ruptured near the Pt electrode and a high resistance state (HRS) can be achieved again if a negative voltage is applied on the Cu electrode in process (3), which can be attributed to the Joule heating effect. Generally, the voltage sweeping in process (1), at which the current suddenly increases over the compliance current, is defined as the V_SET_. On the other hand, there is also a negative voltage sweeping in the process (3) when the current drops abruptly and the resistance switches from LRS to HRS. That voltage value is defined as the V_RESET_. It is clear that standard bipolar resistive switching characteristics can be observed from the I-V curves of CBRAM devices with an IWZOx, IWZOy, IWZOx/IWZOy (x < y) switching layer. Obviously, the I-V curves of an IWZOx/IWZOy (x < y) CBRAM device are exhibited relative to a uniform set and reset process, as shown in Figure 4d. Interestingly, the CBRAM device with IWZOy/IWZOx (x < y) cannot execute the resistive switching behavior, as shown in Figure 4e. The deposition of bilayer IWZO thin film is a continuous process. The oxygen flow increases from 9% to 26% instantly, so that the surface of IWZOx is modified by instantaneous oxygen plasma treatment. The dense and oxygen-rich dielectric layer is formed, resulting in the path of conductive filaments being fixed or unable to be formed between the top and bottom electrode. Based on the aforementioned reason, the function of the IWZOy/IWZOx CBRAM device is a failure, as shown in the schematic model Figure 5.

According to those experimental results, a physical model has been proposed to explain the phenomenon that an excellent resistive switching behavior can be achieved by the stacking of the IWZOx/IWZOy (x < y) switching structure, while the device with a stacking IWZOy/IWZOx (x < y) switching structure cannot be executed the resistive switching behavior (HRS and LRS.) As shown in Figure 5, More Cu ions will be dissociated from the Cu electrode when a positive voltage is applied, and those Cu ions will penetrate into the switching layer (IWZOx) with a large numbers of defects. Therefore, a defect-rich switching layer (upper layer) may be good for keeping Cu ions during the set process (1). However, the enhanced conductive filament near the Pt electrode might also be difficult to be ruptured by the Joule heating effect during the reset process (3). Therefore, a less defective IWZOy switching layer (bottom layer) may introduce a thinner conductive filament that would be easier to be ruptured. As a result, stacking a suitable sequence of bilayer switching may be very helpful to improving the performance of CBRAM devices.

**Figure 6:**
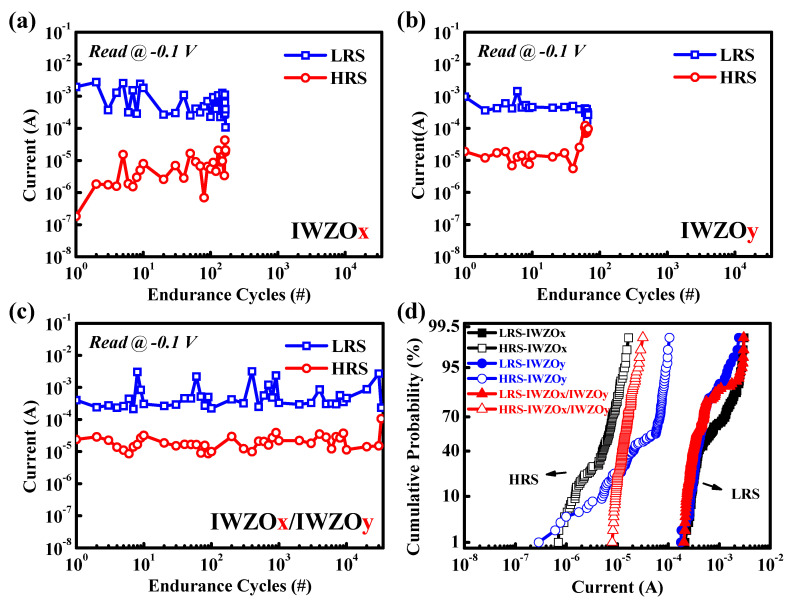
Endurance cycles of CBRAM devices with (**a**) IWZOx, (**b**) IWZOy, and (**c**) IWZOx/y switching layers; (**d**) cumulative distribution for LRS and HRS of CBRAM devices with different switching layers.

**Figure 7:**
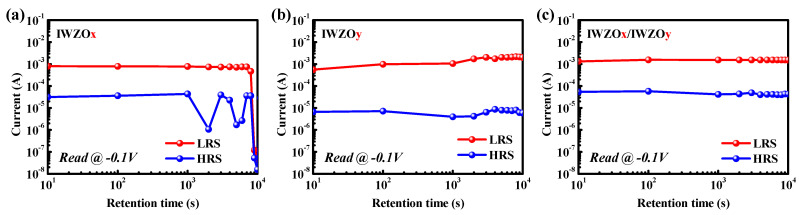
This data retention time of CBRAM devices with (**a**) IWZOx, (**b**) IWZOy, and (**c**) IWZOx/IWZOy switching layer operated at 85 °C.

In order to further investigate the reliability of the CBRAM device, the endurance cycles and cumulative distributions are exhibited in Figure 6. Figure 6a,b shows the endurance cycles of CBRAM with single IWZOx and IWZOy switching layers. It is approaching 200 and 500 cycles, respectively. Notably, the CBRAM device with an IWZOx/IWZOy (x < y) switching structure exhibits a better endurance cycle, which is up to 3 × 10^4^ cycles, and a better cumulative distribution for LRS and HRS when compared with other CBRAM devices with a single switching layer. In addition, Figure 7 shows the data retention time of CBRAM devices with different switching layers at 85 °C. Surprisingly, the CBRAM device with the IWZOx/IWZOy (x < y) switching structure also exhibits an excellent ability to maintain the memory window (LRS/HRS over 10^2^) for 10^4^ s, even at 85 °C. According to those experimental results, it is believed that this specific stacking bilayer IWZO CBRAM might further pave the way for emerging memory applications.

## 4. Conclusions

In this study, the influence of oxygen concentration in IWZO, which was used as the switching layer of CBRAM, was investigated. Compared with single switching layer devices, the CBRAM with a specific stacking sequence of bilayer IWZOx/IWZOy (x < y) exhibits more stable distribution of the resistance state and better endurance (more than 3 × 10^4^ cycles). Meanwhile, the memory window of IWZOx/IWZOy can even be maintained at over 10^4^ s at 85 °C. Those improvements can be attributed to the oxygen vacancy distribution in the switching layer, which may create a suitable environment for conductive filament formation or rupture. Therefore, it is believed that this specific stacking bilayer IWZO CBRAM might further pave the way for emerging memory applications.

## Data Availability

The data presented in this study are available on request from the corresponding author. The data are not publicly available due to privacy.
